# Colorectal Cancer Stem Cells: An Overview of Evolving Methods and Concepts

**DOI:** 10.3390/cancers13235910

**Published:** 2021-11-24

**Authors:** Maria Laura De Angelis, Federica Francescangeli, Ann Zeuner, Marta Baiocchi

**Affiliations:** Department of Oncology and Molecular Medicine, Istituto Superiore di Sanità, Viale Regina Elena 299, 00161 Rome, Italy; marialaura.deangelis@iss.it (M.L.D.A.); federica.francescangeli@iss.it (F.F.); ann.zeuner@iss.it (A.Z.)

**Keywords:** cancer stem cells, colorectal cancer, animal models, in vitro culture, cancer stem cell methods

## Abstract

**Simple Summary:**

In recent years, colorectal cancer stem cells (cCSCs) have been the object of intense investigation for their promise to disclose new aspects of colorectal cancer cell biology, as well as to devise new treatment strategies for colorectal cancer (CRC). However, accumulating studies on cCSCs by complementary technologies have progressively disclosed their plastic nature, i.e., their capability to acquire different phenotypes and/or functions under different circumstances in response to both intrinsic and extrinsic signals. In this review, we aim to recapitulate how a progressive methodological development has contributed to deepening and remodeling the concept of cCSCs over time, up to the present.

**Abstract:**

Colorectal cancer (CRC) represents one of the most deadly cancers worldwide. Colorectal cancer stem cells (cCSCs) are the driving units of CRC initiation and development. After the concept of cCSC was first formulated in 2007, a huge bulk of research has contributed to expanding its definition, from a cell subpopulation defined by a fixed phenotype in a plastic entity modulated by complex interactions with the tumor microenvironment, in which cell position and niche-driven signals hold a prominent role. The wide development of cellular and molecular technologies recent years has been a main driver of advancements in cCSCs research. Here, we will give an overview of the parallel role of technological progress and of theoretical evolution in shaping the concept of cCSCs.

## 1. Introduction

**Figure 1:**
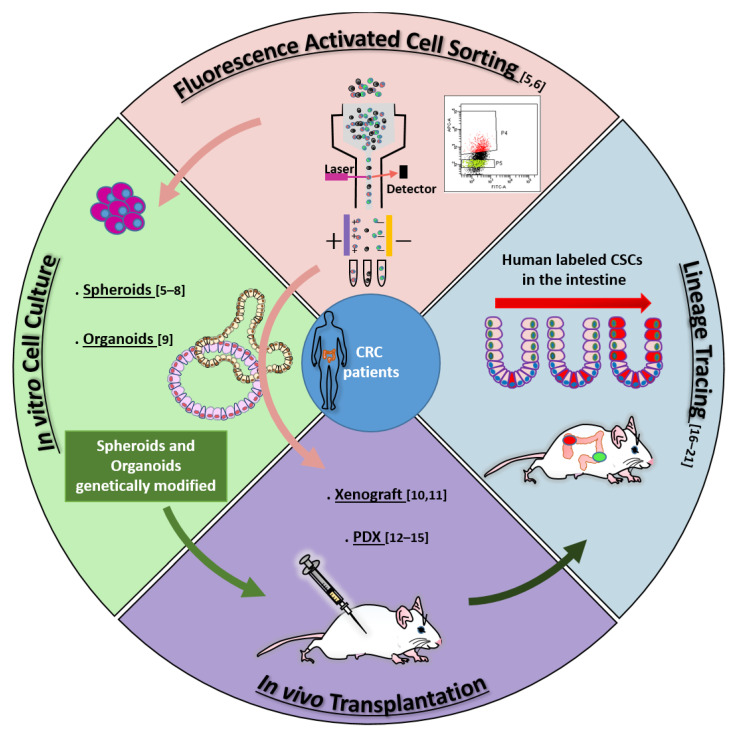
Main methodologies contributing to cCSCs definition: fluorescence-activated cell sorting (FACS) [5,6]; in vitro cell culture [5,6,7,8,9]; in vivo transplantation [10,11,12,13,14,15]; lineage tracing [16,17,18,19,20,21]. Arrows across sectors identify the complementary application of different techniques: For example, identification of cCSCs by cell sorting can be validated by in vitro cell culture and/or by in vivo transplantation assays (pink arrows); Xenografting is often performed with cultured and/or genetically labeled cCSCs (light green arrow); human cCSC lineage tracing is mostly performed by transplanting cCSCs previously genetically manipulated in culture (dark green arrow). Numbers in parentheses indicate references.

**Figure 2:**
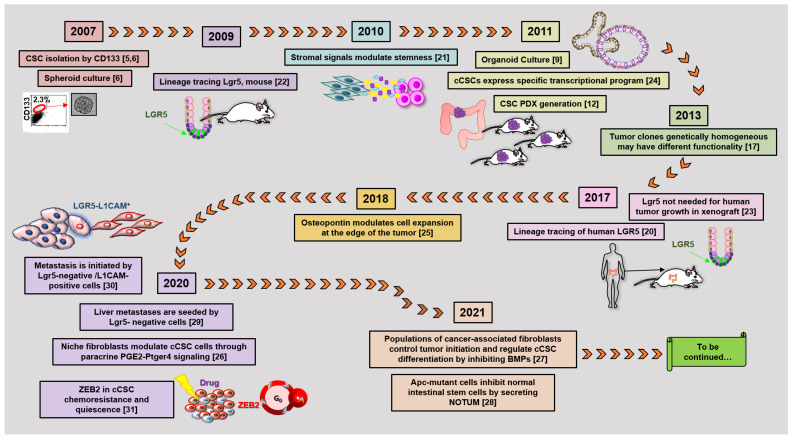
The timetable of landmark studies that have contributed to defining cCSC biological features and functions. Numbers in parentheses indicate references [5,6,9,12,17,20,21,22,23,24,25,26,27,28,29,30,31].

According to the stem cell model, most tumors, including colorectal cancer (CRC), contain a small population of cancer stem cells (CSCs) that are deeply implicated in tumor generation and progression, drug resistance, recurrence, and metastasis [1,2]. The characterization of the molecular and functional features of colorectal CSCs (cCSCs) has thus received intense research efforts in recent years due to its promise to reveal new routes of intervention for tumor and metastasis eradication. Methods and concepts for understanding CSCs biology in solid tumors historically derived from studies on normal hematopoiesis and leukemia. In fact, the CSC concept itself originated from landmark research [3,4] that in the early nineties first showed that leukemia is organized as a hierarchical system, mimicking that of the normal hematopoietic system. The neoplastic cell hierarchy in leukemias develops from a small subset of stem cells able to both self-renew and to give rise to a cascade of more differentiated cells. Notably, the golden standard for the identification of leukemic stem cells (LSCs) was established by extending the definition for normal hemopoietic cells as cells functionally capable of initiating neoplasia into recipient mice. A few years later, the experimental methods that led to LSCs identification were translated to solid tumors in general, and in particular to CRC. During the following years, the expanding range of methodological approaches applied to cCSCs biology has paralleled a continuous evolution of the cCSCs concept, providing a significant example of knowledge advancement about a complex biological issue. The main technical approaches that contributed to cCSCs identification and characterization, in particular of human CRC, are summarized in Figure 1, which also underlines the high degree of intersection of their application. Methods for cCSCs identification can be broadly summarized as (1) cell isolation by fluorescence-activated cell sorting, (2) cell culture-based selection systems, (3) transplantation into recipient animals, and (4) lineage tracing techniques. Here, we will revise the major advancements that led to the development of the current cCSC concept, keeping a historical view on the evolution of technologies that allowed cCSC characterization to the present day (Figure 2).

## 2. cCSC Definition by Experimental Models

### 2.1. Identification of cCSCs through the Use of Surface Markers

Fluorescence-activated cell sorting on the base of CD133 expression (also known as Prominin 1) was used in 2007 by two independent groups [5,6] to identify and isolate for the first time putative cCSCs. By using similar approaches, in fact, the two groups demonstrated that the CD133-positive subpopulation isolated by cell sorting from dissociated patient’s tumors is enriched in xenograft-initiating cells.

The use of CD133 as a stem cell marker is a representative example of an approach inherited by hematopoiesis studies, in which cell membrane markers have long been used to isolate different cell and progenitor subpopulations. Indeed, CD133 was first identified in 1997 as a marker of normal human hematopoietic stem cells [32] and later indicated as a generic marker of endothelial [33] and neural [34] stem cells. In 2003, CD133 was used to identify glioblastoma stem cells [35], opening the way for its future use as a stem cell marker in multiple solid and hematopoietic tumors. 

The efficacy of CD133 as a cCSC marker was shortly thereafter, confirmed by other reports, in particular, Vermeulen et al. demonstrated that CD133-positive cells isolated from patients and grown as spheroids could differentiate in vitro, giving rise to cells belonging to all the main intestinal cell lineages [7]. Lineage tracing experiments in vivo (see below in this review) in turn showed that deregulating β-catenin activity in normal CD133-positive intestinal cells induced neoplastic transformation [36]. However, studies identifying cCSCs through CD133 expression introduced different issues related to cCSC selection from solid cancers based on membrane markers. First, the functional role of cCSC membrane markers is still largely unknown, so that a clear link between their expression and stem cell function can hardly be established. Indeed, analyses of the prognostic value of CD133 expression has given inconsistent results [37,38], and its inhibition had no effect on tumor course [38]. A second issue of concern is that the dissociation of solid tissues may alter the surface density and/or modify the molecular structure of the marker itself, both because dissociation mostly involves the use of proteases and/or because the loss of cell–cell interaction itself can induce reprogramming of membrane–molecule expression. Intrinsic fluctuations of marker expression may also contribute to confusing the outcomes of marker-based CSCs assays. For example, specific mutations in the RAS–RAF axis can alter CD133 expression in CRC cells, independently from their capability to initiate tumors into immunodeficient mice [39]. In addition, cell-state-dependent modifications [40] or glycosylation [41] of CD133 can modify its antibody-binding capability rather than the expression level of the protein itself. Similarly, isolation of cCSCs through CD44, alone or combined with CD166 [42], is complicated by the many alternative splicing-generated CD44 isoforms and by its ubiquitous expression [43]. More recently, the isoform CD44v6 was reported as a strong marker of metastatic cell capability [44]. Interestingly, histological analyses of membrane markers expressed in cells at the edge and in the central zone within human tumors showed that a lower expression of stem cell markers, including CD44 isoforms, CD166, and CD133, by edge cells’ correlates with the infiltrating pattern [37,45]. The authors suggested that downregulation of these molecules may be linked to stage-dependent modulation of cancer cell adhesion capability [37]. Flow cytometry isolation of cCSCs has also involved other stemness-related factors, although not expressed on the cell surface. Among these, aldehyde dehydrogenases (ALDH) are a class of detoxifying enzymes responsible for the oxidation of intracellular aldehydes that hold a particular interest for their potential functional role in stemness [46]. However, studies on ALDH as a CSCs marker in colorectal and other tumors gave conflicting results, likely due to the wide number of ALDH isoforms present in different cell types [47].

More generally, a dynamic modulation of membrane markers of cCSCs can easily be expected to relate to the plastic nature of these cells, as discussed more in detail later in this review. As an example, we have recently described in clonal cCSCs a highly regulated, fluctuating expression of Cripto-1, an extracellular GPI-anchored protein, which correlates with the cell’s variable clonogenic capability [48].

### 2.2. In Vitro Cultures of cCSCs

According to the classical definition, stem cells, either normal or neoplastic, must hold the double functional capability to self-renew and to give rise to a differentiated cellular progeny in the long-term. In light of this concept, appropriate methods for in vitro culture of cCSCs allow assessing both the long-term propagation of tumor-initiating capability (indicative of self-renewal) and a conserved ability to generate differentiated cells. Importantly, culture in selective media can also represent an approach for cCSC isolation from freshly dissociated tissues without prior isolation by marker selection.

The isolation and amplification in vitro of intestinal CSCs inherited the same challenges affecting stem cell cultures of other normal and tumor tissues, and primarily the difficulty of preserving the capability of stem cells to self-renew. The achievements in this field were linked to the progressive identification of components for defined media that paralleled an increasing understanding of the molecular and cellular mediators underlying the stemness state. Importantly, an obvious issue that distinguishes cultures of solid tumor stem cells versus leukemia’s is that adhesion is essential for the former [49] while blood cells are naturally non-adhering. The requirement for adhesion is addressed by different means in the two most diffused intestinal CSC culture methodologies, i.e., spheroid and organoid cultures. 

Spheroids are grown in low-adherence cell culture plasticware, in which, after a few days, dissociated cells from the patient’s tissues generate self-adhering floating clusters that can reach up to 1–2 mm size. Depending on the strain, tubular structures develop within single spheres, associated with the presence of differentiated cells [7,8]. cCSC-enriched spheroid cultures can be expanded long-term, retaining both their xenograft-initiating capability and the potential to generate differentiated cells. Spheroid cultures have allowed the identification and first characterization of cCSCs in early studies [6,7]. 

As mentioned before, spheroid culture in selective media allows direct isolation of cCSCs from dissociated patient tumors. Our group optimized this method, providing a highly efficient workflow for cCSCs isolation from primary tumor fragments, which allowed us to generate a biobank representative of patients’ molecular diversity [8]. A distinctive characteristic of spheroid cultures is that they allow great cell expansion; therefore, spheroid biobanks are particularly convenient for studies requiring a high number of cells, such as high-throughput molecular analyses or drug testing [50]. Ours and other groups have identified different potential cCSC-targeted agents through a spheroid-based platform [48,51,52,53].

Organoid cultures represent an alternative approach for cCSC expansion from patient tissues [54]. Here, the cell requirement for adhesion is met by growing the cells embedded in a basement membrane matrix, usually Matrigel. This system was developed first for normal murine small intestine [55] and then for human tumor intestinal CSCs [9,56]. Organoids preserve a capability to generate tubular, complex structures reproducing the original tumor’s architecture more evidently as compared to spheroids. The use of Matrigel, however, renders the system more expensive and time-consuming, also due to the uneasy release of cells from the embedding matrix. Importantly, patient-derived organoids have allowed generating of biobanks that have proved highly representative of gastrointestinal cancer patients’ response to drugs and radiation [10,11,57,58]. 

Both spheroid and organoid cultures containing human cCSCs can be easily genetically modified, thus offering a frame to analyze the role of known or candidate molecular cancer determinants. To mention a particularly interesting group of studies, the organoid culture system coupled with the CRISPR methodology allows dissecting mutational events underlying tumorigenesis. The sequential introduction of mutations into the *APC*, *SMAD4*, *TP53*, and *KRAS* genes, in fact, lead organoids to reproduce the adenoma-carcinoma transition, disclosing a parallel progressive loss of cell requirement for niche factors [59,60,61]. Recent studies, in turn, have attempted to reconstruct the mutational landscape underlying metastatic tumor capability [62,63,64]. Importantly, cCSCs cultured both with the spheroid and organoid systems can be used to generate xenografts into immunodeficient mice (see Figure 1). Transplantation of cultured and/or genetically modified cultured intestinal CSCs is an approach of the utmost importance in the understanding of cCSC biology, as described in the following sections.

### 2.3. cCSC Transplantation Assays

The assessment of tumor-initiating capability, in vivo, into recipient mice (syngeneic when murine stem cells are tested, immunodeficient for human cell assays) is defined as the golden standard for stemness descending from the historical definition established for putative hematopoietic and leukemic stem cells. In this frame, the only ethically feasible approach to assess the stemness of putative human intestinal CSCs is xenotransplantation into immunodeficient mice. Beginning with the discovery of the spontaneous mutant nude mice carrying T-cell deficiency, increasingly immunodeficient murine strains have become available over time, including the SCID (impaired in T and B cells) and the SCID Beige strain that is further impaired in NK activity. Nowadays, the most used immunodeficient strain in human CSC research is NSG (NOD-*scid* IL2rg^null^), highly deficient in T, B, and NK cell activity [65]. 

Xenografting of human cCSC either from in vitro cultures or from freshly dissociated tumor samples is widely diffused as a stemness assay. Limiting dilution assay in vivo and linear regression analysis, easily performed by the online software ELDA [66], allows quantitative analysis of cCSC content in a cell population of interest, while serial re-transplantation experiments can be used to assess cCSC capability for long-term propagation. Importantly, serial cCSC transplantation demonstrated that different classes of cCSCs in spheroids are functionally heterogeneous, as they can be distinguished in short versus long-term tumor-initiating cells as well as in metastasis-initiating cells [16]. 

Xenografting of tissues or freshly dissociated cells from patient tumors is commonly named patient-derived xenografting. By continuous re-transplantation of xenografts from mouse to mouse, this system allows to propagate and expand cCSCs in vivo, thus bypassing any in vitro culture. Patient-derived xenografts (PDX) have been shown to better preserve the original tumor’s characteristics, as compared to cultured cCSC grafts. However, this system is expensive, laborious, and time-consuming due to the requirement of high numbers of mice and to the slow development of the grafts [67]. 

Human cCSC functional assessment by xenografting is not devoid of limits, the first of which is that the estimate of CSC can be influenced by the recipient mouse strain: more immunodeficient strains may detect a higher frequency of stem cells; such an effect has been reported in detail in melanoma [68]. In addition, microenvironment cells, including vascular, immune, and mesenchymal cells that are not human, while the immune system is by definition impaired in immunodeficient mice. Therefore, the contribution of microenvironment cells to tumor development can hardly be evaluated in xenografts. The use of humanized mice, in which a human hematopoietic/lymphoid system is reconstructed by transplanting hematopoietic cells, allows overcoming some of these limitations [69]. Another issue of concern is that in subcutaneous xenografts—the most feasible and therefore most often used system—and the natural organ location of the tumor is also mismatched; indeed, CSCs of the majority of solid cancers, including colon, do not give rise to metastasis upon subcutaneous grafting [70]. Conversely, orthotopic xenografting of cCSCs into the colon results in metastases to the liver and other target organs, but the method requires technical skill [71]. An alternative system, technically easier, is grafting cCSCs into the spleen, whose vascularization leads directly to the liver [71]. This system, however, reproduces only partially the whole process of cellular metastasization, from the primary tumor to the target organ. 

Despite these caveats, panels of both cultured cCSC xenografts and PDX have revealed an important therapy-predicting capability [12,13,14,15], while cCSC transplantation has contributed to collecting a whole bulk of information on human cCSCs biology. Importantly, CSCs xenografting represents a unique approach for human CSC lineage tracing (See Figure 1). 

### 2.4. Lineage Tracing of cCSC

In general, lineage tracing methods consist in following the development of a given progenitor/stem cell progeny on the basis of a morphological feature or a dye or a molecular/genetic marker that is conserved and transmitted during the developmental process [72,73]. The simplest cell labeling systems consist in staining the cells with supervital colored or fluorescent dyes. However, the development of genetic manipulation techniques, coupled with the increasing availability of high-throughput sequencing methods, are generating an ever-expanding genetic lineage tracing toolbox. Among these, the most direct system is to introduce into the cells of interest a gene for a colored (such as β-galactosidase or alkaline phosphatase) or fluorescent (such as EGFP, RFP, EYFP, tdTomato, and others) or light-emitting marker. An advancement of this approach consists in using conditionally expressed markers: this is obtained by Cre-*Lox* and derived systems, where Cre is a P1 bacteriophage-derived DNA recombinase, and *LoxPs* are its recognition sites. In the Cre-*Lox* tracing systems, the expression of the labeling gene is blocked by a stop cassette flanked by two *LoxP* sites. Concurrently, the cells also carry a *Cre*-recombinase gene, which is activated by a tissue- or cell-stage-specific promoter; therefore, the stop cassette is excised, allowing the expression of the marker gene only in the tissue or the subset of cells that activate the chosen specific promoter. In inducible Cre-*Lox* systems, the expression of *Cre* is further controlled by an inducible element, for example, a tamoxifen-responsive sequence. A pulse of the drug triggers the activation of the labeling gene in the cell population of interest at a time point of choice. This allows, for example, to distinguish stem cells, able to self-renew for prolonged periods of time, from downstream progenitors that may express the same marker but get exhausted over time due to their limited proliferative capability. 

Later technologies include multicolor systems such as Confetti and Brainbow, in which conditional, inducible Cre-*Lox* systems induce different fluorescent labeling at random in single cells, allowing to follow the destiny of several individual clones within a tissue. Up to four and ninety different fluorescent wavelengths are generated by the Confetti and Brainbow systems, respectively [73]. Random multifluorescence labeling systems have been widely used to follow specific marker gene-expressing cells, including *Lgr5* (see below), but they are now increasingly being adapted to marker-free labeling. Importantly, dye-free labeling systems have also seen an expanded use in recent years, taking advantage of the high sequencing capability provided by high-throughput technologies: it is thus possible to genetically barcode cells by analyzing the propagation of short sequences/mutations introduced either by lentiviral vectors [16,17] or more recently by CRISPR/Cas9 genome editing [18]. Individual clones are then identified by high-throughput sequencing. Finally, non-labeling approaches based on spontaneous randomly occurring mutations have also been used to follow CRC clonal growth by taking advantage of mitochondrial mutations and/or by single nucleotide/copy number variations (SNV/CNV) [18,19]. Lineage tracing can allow following natural cell development into its own whole organism: in the mouse model, this is achieved by crossing strains carrying loxed labeling gene(s) with strains carrying inducible and/or conditional Cre recombinase. Conversely, tracing of human cells requires the manipulation of cells in vitro, followed by xenotransplantation in an immunodeficient recipient animal. In general, either approach has its own specific limitations, since, as mentioned before, murine cancer models do not fully reproduce the human pathogenesis, while xenotransplanted human cells suffer from microenvironment mismatch. Nevertheless, both murine and human lineage tracing technologies have given landmark information on CSC biology in intestinal adenoma/carcinoma. 

Lineage tracing of GFP-labeled CD133 cells in mice allowed confirming their location at the base of the crypt and their capability to give rise to all the intestinal epithelium [36]. In addition, CD133+ cells exhibited massive amplification, generating neoplastic tissue within the intestine upon Cre-dependent, promoter-specific activation of mutant β-catenin, thus demonstrating their stem cell nature [36]. 

A major contribution of lineage tracing, however, has been in the establishment and progressive definition of the role of Lgr5+ as a stem cell marker in both normal and neoplastic intestinal stem cells. Lgr5 (leucine-rich repeat-containing receptor) is a transmembrane receptor involved in the modulation of the canonical Wnt signaling pathway. As the Wnt pathway is a primary driver of normal and neoplastic intestinal cell development [74], Lgr5 holds the features of a functional marker in intestinal cancer development. However, early studies on Lgr5 role in intestinal tumorigenesis were largely based on lineage tracing, as the scarce availability of efficient commercial antibodies against Lgr5 traditionally hampered the isolation of Lgr5+ cells by FACS. 

Labeled Lgr5+ cells were first demonstrated to be capable of giving rise to all the intestinal cell types in the normal mouse intestine by using a tamoxifen-inducible *GFP* integrated into the *Lgr5* locus [75]. Shortly thereafter, the same group also showed that conditional deletion of Apc in the same cells induces them to proliferate and generate adenomas invading the crypt [76]. In addition, in *Apc*-mutant mice, random labeling of single Lgr5+ cells by a tamoxifen-activated multicolor Confetti system allowed to visualize adenomatous clones of different colors growing from the base to the upper edge of the intestinal crypt [22]. Lineage tracing of human cCSC was later achieved by labeling Lgr5+ cells into human organoids upon xenotransplantation: EGFP-labeled Lgr5+ cells proved to be able to initiate xenografts and to differentiate into the main intestinal lineages [20]. Altogether, these studies established Lgr5 as a common marker for both normal and tumor intestinal stem cells.

## 3. Merging Methodologies and Evolving Concepts: cCSC Plasticity and the Niche

The different approaches discussed above have altogether contributed to defining cCSC as a cell subpopulation driving initiation, development, and growth of CRC. Nevertheless, parallel research has challenged the concept that a fixed phenotype could be attributed to such population(s). Early observations indeed indicated that cells not expressing specific markers might undertake a functional stem cell role, at least under stress circumstances. Among these, Shmelkov et al. followed intestinal murine CD133 expression by a Lac-Z reporter showing that both CD133+ and CD133– cells can initiate metastases into immunodeficient mice [77]. Later, Lgr5+ cells were found dispensable for adenoma formation after irradiation in Apc-mice [78], while a population of keratin-19 (KRT19)-positive, Lgr5-negative cells were shown to be responsible for cancer tissue regrowth following irradiation in mice [79]. More recently, de Sousa et al. analyzed the effects of Lgr5+ cell removal through an inducible diphtheria toxin in a model of murine colon cancer. After killing Lgr5+ cells by toxin activation, tumor growth was not impaired, indicating that other cell type(s) could take over a stem cell function [80]. Similar results were shown in human CSCs, by xenotransplanting organoids carrying an inducible gene for caspase 9 inserted into the *Lgr5* locus. Elimination of Lgr5+ cells by caspase activation blocked tumor growth. Upon removal of the killing stimulus, however, tumor regrowth was driven by a population of Lgr5−/KRT20 positive cells, which regenerated Lgr5+ cells [23]. 

**Figure 3:**
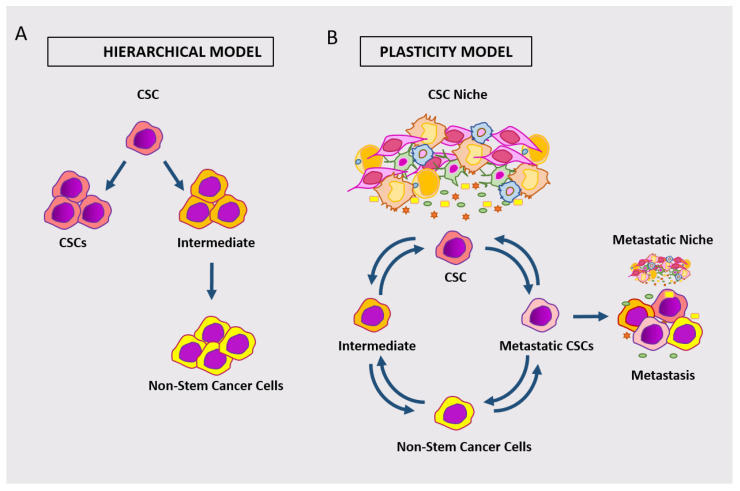
Schematic representation of the hierarchical model of cCSC (**A**) versus the model of plastic cCSC (**B**). According to the plastic model, different classes of cancer cells can dynamically take on stem cell phenotypes/functions.

The observations that stem cell marker-expressing cells may not be the unique drivers of adenoma/carcinoma points to the issue of stem cell plasticity. This concept includes both the capability of intestinal CSCs to acquire different phenotypes and the potential of different tumor cell subpopulations to take on stem cells function under different circumstances [1,2,81]. A scheme comparing the hierarchical model of cCSC versus the emerging model of plastic cCSC is shown in Figure 3.

The plasticity of cCSCs was also supported by other studies tracking stem cells on the base of Wnt activation state rather than through stem cell marker expression. In fact, tracing human xenografted cCSCs by means of a Wnt-dependent GFP reporter showed that cCSCs driving tumor expansion are located at the edge of the tumor. In this system, signals from surrounding stromal cells proved to be instrumental in modulating Wnt activation levels in tumor cells, pointing to a role for stroma in inducing cCSC function [21]. In turn, constitutive NF-kB activation was reported to enhance Wnt activation and stem cell marker expression in mouse crypt cells [82]. More recent phenotype-independent tracking experiments keep extending this concept. Phenotype-independent lineage tracing of human cCSCs confirmed that clonal expansion in colon cancer xenografts mostly arises at the leading edge and that cell position is a main driver of clonal competition during tumor development [83]. Other studies used an improved multicolor marker-independent tracing system (RGB/LeGO) to follow long-term clonal dynamics within xenografts, showing a correlation between clone size and proximity to the edge. These observations further support a persisting role of tumor geometry and cell position in orchestrating clonal competition during tumor growth [84]. 

The plastic nature of intestinal CSCs is also consistent with the knowledge that genetically homogeneous cells within the tumor can take over different functions, as demonstrated by clonal analysis of expanding human cCSC clones in xenografts by lentiviral marking [17] and by ultra-deep whole-genome sequencing tracking SNV/CNV [18]. In addition, cCSCs are known to express specific transcriptional programs [24,85,86], and a recent report has shown that high levels of ribosomal activity and protein synthesis individuate cCSCs independently from their specific mutational landscape [87]. It is noteworthy that genetic studies on patient’s adenoma and CRC tissues collectively indicate that driver mutations are mostly established early in the first stages of tumorigenesis, while limited functional mutational divergence is added during tumor development, thus strengthening the idea that non-genetic events are the main determinants of intestinal CSCs function during tumor development [88,89,90]. 

The concept that cCSC plasticity may be related to cell location points to the role of the so-called tumor niche in instructing cCSC behavior. In fact, cells at the tumor’s edge reside in close proximity to stromal cells, thus being exposed to stroma-derived signals. Early studies had actually described an instructive role of the niche and of niche-secreted factors, including WNTs, R-spondins, and BMP-inhibitors, in influencing the fate of intestinal CSCs [82,91,92,93,94]. In recent years, research on tumor niche has been expanding, disclosing the complexity of the crosstalk that orchestrates the plastic features of cCSCs. In this frame, Lenos et al. recently described a relevant role of stroma-secreted osteopontin as an inducer of cell expansion at the edge of the tumor [25]. Another report identified a specific subpopulation of fibroblasts in the mesenchymal tumor niche, which controls tumor-initiating cells through paracrine PGE2 (Prostaglandin E2)-Ptger4 signaling [26]. In turn, polarized populations of cancer-associated fibroblasts regulate cCSC differentiation and cancer progression by balanced inhibition of BMPs by GREM1 [27]. 

In this frame, transient epigenetic modifications, including variation in DNA methylation, histone modification, and chromatin accessibility, certainly contribute to sustaining CRC cell stemness [95,96,97]. Several epigenetic mechanisms capable of affecting CRC cell stemness, including but not limited to Wnt pathway activation/inactivation, have been described [98,99,100,101,102,103]. In this context, cell position-related epigenetic modulation likely holds a particularly relevant role in cCSC plasticity [95,96,97]. For example, tumor cells located at the edge or in central areas of the tumor are exposed to varying concentrations of metabolites and oxygen, which can modulate histone modifications and DNA methylation [96]. Stroma-secreted factors acting on cCSC plasticity, among which TGFβ, in turn, are able to induce cell epigenetic modifications [104]. Novel methods for single-cell epigenetic analyses, aided by developing computational systems, are now beginning to shed light on inherited epigenetic states of cellular lineages within CRC [105,106].

Further complexity is added to the picture by the finding that cCSCs are able to deliver inhibitory signals to normal intestinal cells, both directly and by inducing stromal cells to secrete specific factors. In this frame, two studies recently described the capability of Apc-mutated adenoma cells to inhibit stem cell activity of non-mutated cells within the same crypt and adjacent crypts through secretion of soluble Wnt antagonists [107]. Among these factors, a prominent role is played by NOTUM, whose pharmacological inhibition blocks adenoma formation [28]. Other important information has been provided by an innovative tracking system, the Confetti-derived Red2Onco, by which oncogenes such as mutated *KRAS* or *PI3K* are inserted only in RFP+ tumor cells. By allowing tracking of separately normal and mutated intestinal cells, this system has revealed that oncogene-driven signals from mutated cells induce apoptosis and differentiation of surrounding normal intestinal stem cells, both directly and by instructing surrounding stromal cells to secrete inhibitory factors [108]. 

The plasticity of cCSCs holds a particular interest in view of their capability to initiate metastasis. Several recent studies have described variable stem cell marker expression in metastasis-initiating cCSCs. For example, in the study by de Sousa e Melo already mentioned, while the selective elimination of Lgr5+ cells did not lead to tumor growth stopping, metastasis initiation in the liver was delayed until the Lgr5+ cell re-emerged [80]. By using intravital microscopy on xenotransplanted organoids carrying an inducible Lgr5-EGFP-Confetti, Fumagalli et al. followed metastases seeding and initiation, observing that liver metastases are seeded by Lgr5− cells, although Lgr5 positivity and stem cell marker expression re-emerges in growing metastases [29]. Consistently, Ganesh et al. showed that patients’ metastases are initiated by cells overexpressing L1CAM+, that do not concurrently express Lgr5 [30]. Altogether, a picture thus emerges, in which Lgr5 expression is downregulated at some stages of dissemination/seeding, to be then re-expressed during metastasis growth into target organs. Low expression of stem cell markers of metastasis-initiating cCSCs is indeed shared by budding cells at the edge of the tumor [109] and by cCSCs circulating in the blood flow, i.e., putative migrating cells at metastasis target organs [110,111,112,113]. The plasticity of metastasis-initiating/circulating cCSCs has been put into relationship with the transition of cCSCs into quiescent/drug-resistant states [31,114], and involves at least at some stages EMT (epithelial to mesenchymal transition, reviewed in [112]). This capability of cCSCs to downregulate epithelial and/or stem cell markers to take on mesenchymal features is driven by specific transcription factors, including SNAI1, SNAI2, ZEB1, ZEB2, and TWIST1 [112,115,116]. The crosstalk between tumor and stromal cells holds a role of utmost importance in CRC and in cCSCs, in particular through TGFβ signaling [62,63,117]. Finally, other mechanisms of stromal/tumor cell modulating metastatic cCSCs are emerging: a recent report details the reciprocal reinforcement between visceral adipose stromal cells and metastatic CD44v6-positive cCSCs, sustained by adipose cell-secreted IL-6 and HGF, and by neurotropin produced by CD44v6+ cells [118].

## 4. Conclusions and Future Perspectives

Taken together, most recent studies have converged to redefine CRC cell stemness, from the permanent feature of a restricted tumor cell subpopulation to a function that can be undertaken by different cell types under different circumstances. Such a function is modulated by a series of factors awaiting further dissection, but definitely including signals both intrinsic and from the microenvironment [1,2]. Spatial constraints likely also contribute to influencing the clonal development of CRC [119]. It has been proposed that different parameters may dictate clone competition during different developmental phases of tumors, arguing that space constraints may be determinant in leukemia developing into the bone marrow, while stromal signals may have a prevalent role in solid tumors [97]. Even within the same tumor, different parameters may acquire or lose relevance during development: Regarding intestinal cancer, it is easy to hypothesize that cell clonogenicity and stemness may be differently regulated during the initial adenomatous phase, within the restricted crypt environment, and later on, at the expanding invasive edge of carcinoma. Several pieces of evidence indeed indicate that different clonal selection determinants act in adenoma as compared to carcinoma [120,121]. Altogether, the picture of intestinal CSCs represents a fast developing, challenging biological issue.

The wide array of methodological advancements of these years is increasing our knowledge of the cellular events taking place in CRC at a fast speed [122]. New cell culture methods are progressively extending into sophisticated engineering methodologies, among which biomimetic scaffolds and organs on chips [123,124]. Single-cell microfluidics are already contributing to the characterization of circulating cCSCs, and begin to find application in tissue-dissociated cells [97]. Other important developments of in vitro methods aim to reconstruct the contribution of tumor microenvironment components, including co-culture systems of organoids with mesenchymal cells [125] or lymphocytes [126]. Intravital microscopy allows visualizing cells within a whole organ [29,127]. Lineage tracing is increasingly taking advantage by developing high-throughput sequencing techniques [73], allowing unsupervised tagging by lentivirus or by CRISPR, or even by following naturally occurring mutations [128]. Advanced single-cell technologies [129,130], including genome sequencing [131] and scRNA analysis [132,133], are already contributing to colorectal CRC and niche studies [105,134,135] and are expected to gain further strength in the very near future [97]. 

As a final note, the growing impact of computational methods in research on cancer cell biology, and CSCs in particular, deserves a special mention. The analysis and management of the enormous amount of data generated by multi-omic techniques are in fact made possible only by the continuous development of dedicated algorithms, and most of the recent studies mentioned in this review have heavily taken advantage of increasingly sophisticated computational approaches (see for example [84,88,106,107,132,136]). Recent applications include meta-approaches able to integrate data derived from multiple different analytical tools, such as scDNA- and scRNA-sequencing [97]. Complex systems of lineage tracing reconstruction have been developed, such as the so-called pseudotime projection analyses, able to elaborate cell lineage developmental trajectories based on scRNA expression patterns [128]. Similarly, genetic lineage tracing by scDNA-seq allows the reconstruction of spatial models of clonal development in solid cancers [88,130].

Self-training artificial intelligence (AI) tools, in particular, machine learning (ML) and deep learning (DL), have demonstrated an exceptional power in individuating patterns within wide datasets and are currently not only applied to cancer development research but also evaluated for clinical classification and decision-making [137,138]. The specific capability of DL to discriminate and classify images, in particular, surpassed that of humans in 2005 [138], and it is now widely used to analyze large-size imaging datasets. Most importantly, it is also generating breakthrough innovative imaging technologies, among which label-free cell recognition systems, able to predict fluorescent labels in unlabeled microscopy images [139] or ghost cytometry, that allow sorting of cells on the base of marker-free, image-free cell morphology pattern analysis [140].

In conclusion, it is easy to prophesize that such a wide range of fast-developing methodologies are destined to enlighten an increasing complexity of the cCSCs model in the very near future.
